# Accurate locomotor activity profiles of group-housed mice derived from home cage monitoring data

**DOI:** 10.3389/fnins.2024.1456307

**Published:** 2024-09-20

**Authors:** Rongwan Sun, Marie-Christin Gaerz, Christian Oeing, Knut Mai, Sebastian Brachs

**Affiliations:** ^1^Department of Endocrinology and Metabolism, Charité—Universitätsmedizin Berlin, Corporate member of Freie Universität Berlin and Humboldt-Universität zu Berlin, Berlin, Germany; ^2^German Centre for Cardiovascular Research (DZHK), Berlin, Germany; ^3^Department of Cardiology, Angiology and Intensive Care Medicine, Deutsches Herzzentrum der Charité (DHZC)—Campus Virchow-Klinikum, Berlin, Germany; ^4^Department of Human Nutrition, German Institute of Human Nutrition (DIfE) Potsdam-Rehbruecke, Nuthetal, Germany; ^5^NutriAct-Competence Cluster Nutrition Research Berlin-Potsdam, Nuthetal, Germany; ^6^German Center for Diabetes Research (DZD e.V.), Neuherberg, Germany

**Keywords:** activity measurements, circadian profiles, continuous home cage monitoring, metabolism, Alzheimer’s disease, non-invasive, digital ventilated cage

## Abstract

**Introduction:**

Holistic phenotyping of rodent models is increasing, with a growing awareness of the 3Rs and the fact that specialized experimental setups can also impose artificial restrictions. Activity is an important parameter for almost all basic and applied research areas involving laboratory animals. Locomotor activity, the main form of energy expenditure, influences metabolic rate, muscle mass, and body weight and is frequently investigated in metabolic disease research. Additionally, it serves as an indicator of animal welfare in therapeutic, pharmacological, and toxicological studies. Thus, accurate and effective measurement of activity is crucial. However, conventional monitoring systems often alter the housing environment and require handling, which can introduce artificial interference and lead to measurement inaccuracies.

**Methods:**

Our study focused on evaluating circadian activity profiles derived from the DVC and comparing them with conventional activity measurements to validate them statistically and assess their reproducibility. We utilized data from metabolic studies, an Alzheimer’s disease model known for increased activity, and included DVC monitoring in a project investigating treatment effects on activity in a type-1-like diabetes model.

**Results:**

The DVC data yielded robust, scientifically accurate, and consistent circadian profiles from group-housed mice, which is particularly advantageous for longitudinal experiments. The activity profiles from both systems were fully comparable, providing matching profiles. Using DVC monitoring, we confirmed the hyperactivity phenotype in an AD model and reproduced a decline in activity in type-1-like diabetes model.

**Discussion:**

In our work, we derived robust circadian activity profiles from the DVC data of group-housed mice, which were scientifically accurate, reproducible and comparable to another activity measurement. This approach can not only improve animal welfare according to the 3R principles but can also be implement in high-throughput longitudinal studies. Furthermore, we discuss the advantages and limitations of DVC activity measurements to highlight its potential and avoid confounders.

## 1 Introduction

Metabolic diseases such as obesity, insulin resistance, metabolic syndrome, and type-2 diabetes (T2D) are becoming increasingly widespread and represent a global health problem of pandemic dimension (Collaboration NCDRF, 2017). The prevalence of all metabolic diseases increased from 2000 to 2019, with the largest increases occurring in high sociodemographic index countries (Chew et al., 2023). Metabolic parameters often provide information about the health state and can enable early lifestyle changes and the avoidance of risk factors to prevent such diseases or their progression (VanWormer et al., 2017; Handelsman et al., 2023). Persistent overnutrition in combination with reduced physical activity promotes imbalance in energy metabolism, which leads to obesity (Jehan et al., 2020). Thus, locomotor activity is a key factor for metabolism and an important measure in animal experiments, but also in many other research areas, e.g., neuroscience (Caggiano et al., 2018; Hughes and Piggins, 2012; Kemoun et al., 2010; Alonso-Frech et al., 2011), behavioral psychology (Klein et al., 2022; Reyes-Molina et al., 2022; Wermelinger Ávila et al., 2022), development (Ingebretson and Masino, 2013; Hallal et al., 2006), disease treatment (Perse, 2021; Latimer-Cheung et al., 2013; Kodama et al., 2007; Carlson et al., 2014), parasitology (Guimarães et al., 2021; Brachs and Haas, 2008), pharmacology (Kelley, 1993; Niederberger and Parnham, 2021), toxicology (Moser et al., 2016; Mac Phail et al., 1989), and environmental science (Makaras et al., 2020).

Pharmacological and toxicological studies, in which a compound or treatment reduces wellbeing of animals, often result also in reduced activity (Nikodijevic et al., 1993; Terry et al., 2023). For instance, decreased nocturnal activity was reported in animals treated with cisplatin (Dakup et al., 2018; Perse, 2021). Similarly, in neuroscience, genotypes or disease models affect activity patterns and behavior and vice versa. Moreover, activity also plays a role in many neurological diseases. In Parkinson’s disease (PD), physical activity is impaired during the course of the disease, and in addition, many of the patients’ symptoms can also be improved through activity (Fayyaz et al., 2018). Appropriate physical activity can also alleviate symptoms of depression, anxiety, and distress (Zhang et al., 2024; Singh et al., 2023). Thus, the importance of activity assessment has far-reaching implications for the treatment of disease, and more accurate measurements of activity are essential, e.g., for studies of PD models. Furthermore, we revealed increased nocturnal activity in an Alzheimer’s disease (AD) mouse model (APP23) as a specific feature (Schreyer et al., 2021). Overall, activity is therefore an important measure, not only in animal experiments. Additionally, it can be used as a scoring parameter for animal welfare in disease models, including pharmacological and other therapeutic studies. Such an approach can easily be integrated into routine severity assessments. For example, large-scale home cage activity monitoring has been used to assess severity in a dextran sulfate sodium-induced colitis model with an accuracy equivalent to clinical gold standard parameters (Zentrich et al., 2021).

Many different types of devices exist to measure activity of laboratory mice, including Sable Systems Promethion (Soto et al., 2019), Columbus Instruments CLAMS/OxyMax (Soto et al., 2019), Tecniplast DVC (Digital Ventilated Cage system) (Iannello, 2019), TSE systems LabMaster/PhenoMaster (Frenz et al., 1995), and open field activity monitoring system (Tatem et al., 2014) or video-based home cage recording systems (Salem et al., 2024) often combined with artificial intelligence for evaluation (Kopaczka et al., 2019). However, most systems assess a variety of parameters (like indirect calorimetry, etc.) alongside with activity and are therefore not intended for group-house mice. These systems are highly sophisticated, but also expensive and, thus, are inadequate for high-throughput analyses, whereas the DVC is more cost-effective, especially monitoring the animal husbandry in a daily routine. Those systems are often only used for short-term analyses under specific conditions, but animals need further handling, which affects their circadian rhythmicity, behavior, and activity itself. The DVC is an automated home cage monitoring system based on electromagnetic waves to detect cage activity, bedding moisture, and water/diet supply 24/7 in real time of group-house mice in their home cage (Klein et al., 2022), minimizing direct handling and providing animal (welfare) information to reduce operator actions. All continuous data are recorded and can be analyzed directly or retrospectively.

Analyzing circadian rhythms and motor activity is important for accurate biological, behavioral, metabolic, and neuroscience studies (Kamei et al., 1994; Hillar et al., 2018; Stojakovic et al., 2018). In addition, the 3R principles by Russell and Burch (1960) demand lessen any kind of burden and distress. Therefore, we evaluated whether the continuous data recorded in the home cages of group-housed mice could provide qualitative activity profiles while minimizing interaction with the mice and refining their experimental conditions. In addition to the short-term measurements, we also explored the long-term use case to answer whether such activity measurements are beneficial and consistent in longitudinal studies on energy metabolism. Given the limitations in infrastructure, systems, and funding, this approach might help to address shortcomings related to activity measurements, e.g., in behavioral studies. In this study, we first processed and examined the activity data of group-housed mice at individual time points. Next, we compared activity profiles of the same animals using both a well-established LabMaster system versus DVC’s continuous data recordings. Furthermore, we reinvestigated a previously published activity phenotype in the AD mouse model APP23 and verified it using the continuous group-housed data. Finally, we evaluated the treatment effect on activity in a Streptozotocin (STZ)-induced type-1-like diabetes model.

## 2 Materials and methods

### 2.1 Animals

Animal experiments complied with institutional and national ethical guidelines according to European directive 2010/63/EU as well as the 3Rs principle and ARRIVE guidelines and were conducted according to internationally accepted standards. All animal experiments, of which data were previously collected and were reanalyzed for this study, were reviewed and approved by Landesamt für Gesundheit und Soziales Berlin (LAGeSo: G0074/16, G0219/17, G0104/20). DVC data were recorded in home cages in animals maintained for final experiments, approved by LAGeSo (T0180/16) and Forschungseinrichtung für Experimentelle Medizin (FEM: T-CH19/21) at Charité—Universitätsmedizin Berlin.

### 2.2 Cage and husbandry conditions

For this study, we reanalyzed and/or recorded data from wild-type (WT), knockout (KO), and transgenic (TG) APP23 mice. All mice were allocated as littermates to genotype-specific groups, predominantly pair-housed but not mixed per cage after weaning. They were fed a standard maintenance diet (STD, Rat/Mouse-Maintenance V1124-300, Ssniff) or western diet (WD: high-fat diet (60% kcal fat) D12492, Research Diets via Brogaarden, plus 6% sucrose in drinking water) as indicated. Mice were maintained in standard individually ventilated cages with a 12 h light/dark cycle at 22 ± 2°C, a relative humidity of 55 ± 15%, and *ad libitum* access to diet and drinking water until the final experiments. As enrichment, cages were supplied with transparent tunnels, translucent red houses, and the same amount of bite sticks, nesting material, and bedding (500 mL), which was changed weekly on Monday to ensure standardized activity measurement. All conditions for comparisons between genotypes were kept identical.

### 2.3 Activity measurements

#### 2.3.1 LabMaster

For these measurements, a LabMaster from TSE systems (Bad Homburg, Germany) was used, in which mice were analyzed single-housed using the same cage system as in husbandry with red translucent houses, not interfering with activity counts but without other enrichment, for indirect calorimetry as well as activity monitoring. The LabMaster measurements are a standard procedure in our phenotyping pipeline to assess indirect calorimetry and activity of various mouse models (Schreyer et al., 2021; Brachs et al., 2018). All mice were familiarized with the device’s special water bottles in their home cage setting for 24 h 1 week before analysis. Mice were assigned to an individual LabMaster cage in the morning, and measurements were started for 48 h under maintenance of the respective diet. After an approximately 9 h adaption phase, data were evaluated over the next 36 h, including two night phases (6 p.m. to 6 a.m.) as average and one day phase (6 a.m. to 6 p.m.). After measurements, mice were regrouped in their home cages. From the cage data, we calculated mean locomotor activity (XT + YT) in counts per hour and total differential distance (DistD) in cm per hour for each mouse.

#### 2.3.2 DVC monitoring

**Figure 1:**
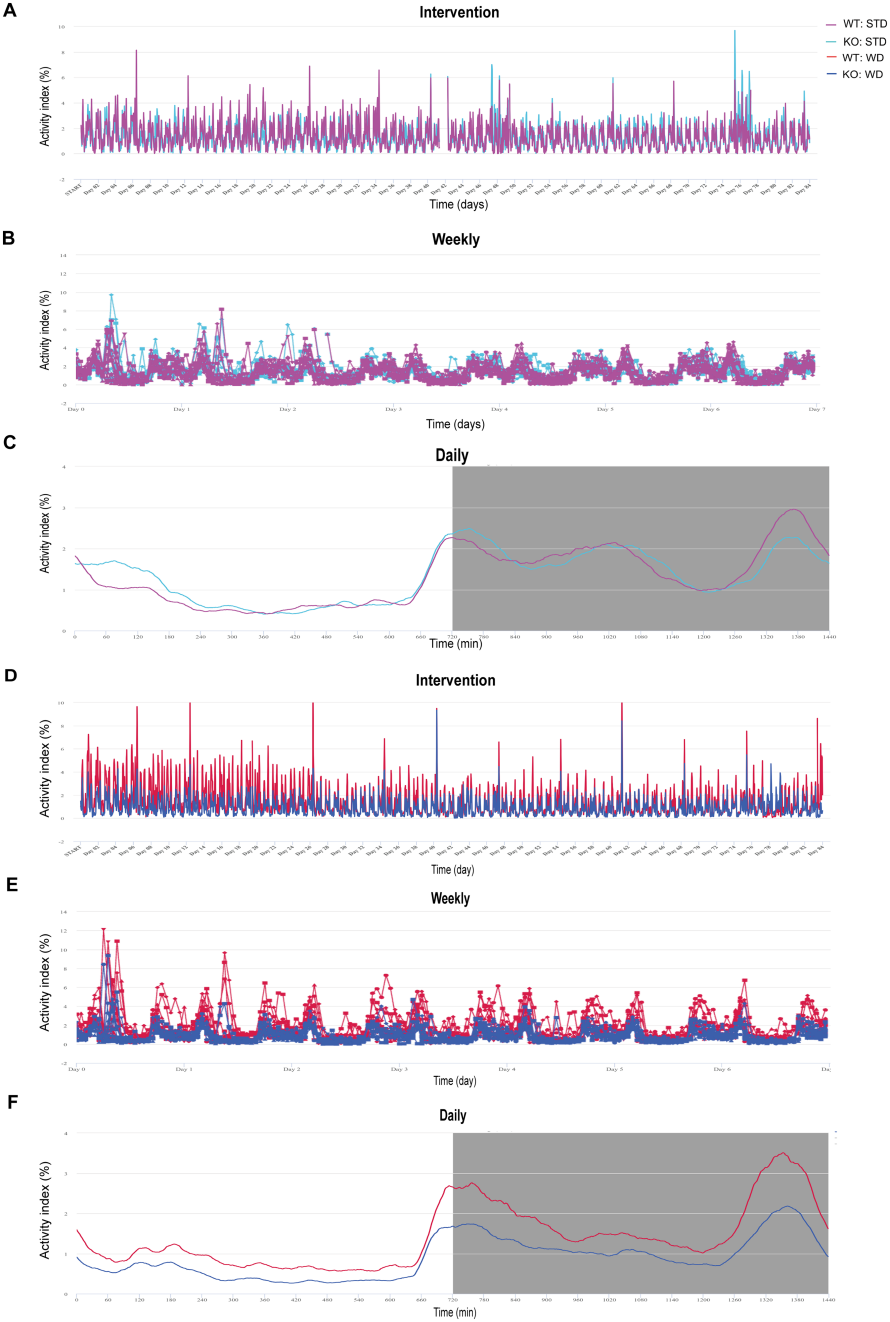
Illustration of activity data from group-housed, genotype-separated WT and KO mice fed STD or WD monitored in the DVC and visualized by the Analytics platform. **(A)** Activity index curves of DVC measurements of STD-fed mice over 82 days. **(B)** Activity as weekly average. **(C)** Mean daily activity averaged over all days. **(D–F)** Analytics visualization of time courses of WD-fed WT and KO mice analog to **(A–C)**. Data are presented on a cage base **(A,B,D,E)** per day **(A,D)** as weekly time course averaging the 12 weeks of intervention **(B,E)** or daily mean as circadian rhythm **(C,F)** in Analytics. Night phases are gray-shaded **(C,F)**. KO, knockout; STD, standard maintenance diet; WD, western diet; WT, wild type.

DVC is a home cage monitoring system based on capacitance sensing technology (CST) non-invasively tracking electrode data of each cage in real time. Under each cage, a sensor board with 12 electrodes is installed sensing electrical changes once every 0.25 s (Iannello, 2019). Measurement of the Animal Activity Index is detailed in Iannello (2019). Briefly, the different electrical capacitance was transformed computationally into the locomotion index, which reflects the activity density of mice. Data are continuously monitored and stored for each cage 24/7 automatically. The activity data were collected and can be visualized using the cloud-based platform Tecniplast DVC Analytics. Data are visualized as monthly, weekly, and daily activity in Figures 1A–C.

From Analytics, activity index data per cage was exported to Excel and processed for statistical analyses, interpretation, scientific visualization, and comparisons with LabMaster activity data. To calculate “individual” mouse activity, the recorded intra-cage activity index as hourly mean was exported and downloaded. Data were further processed in Excel. The time courses and sensor sample readings were compared between the groups, considering the size of each group, and checked for irregularities or highly deviating values or errors such as changeover of winter/summer time, for which the time sequence had to be rearranged. Mean hourly cage activity was averaged by the number of mice in the respective cage, sorted by day and night phase and these mean activity values per mouse were used for further evaluation. To analyze movement distance, the mean animal tracking distance was calculated in cm per hour for each single-housed mouse from WT and KO experiments, averaged over 6 days from the week before the LabMaster measurements.

For scientific DVC analyses, mice were predominantly pair-housed, strictly separated by genotype or treatment, as a mixed cage would not allow calculating activity signals to a single individual. For standardization, bedding (bedding and nesting material) was partially changed weekly on Monday, while cage lid and body with mouse house, tunnel, and bite material were continued. We excluded the day of cage change from activity analysis, not to incorporate this noisy day phase due to exploration behavior of the mice in our data. An exception was the STZ study, for which we defined two regular cage changes per week (on Monday and Thursday) due to the strongly increasing urination caused by STZ treatment. To obtain comparable measurements, this was also carried out for the control group, and data of all days were included in analyses, accepting the increased activity due to the cage changes. Mice of all cohorts were maintained in the DVC until finally sacrificed for cell/organ harvesting, except for the 48 h LabMaster measurements at the end of each intervention.

### 2.4 Mouse models

In this study, we examined data from individual-housed mice from LabMaster measurements and group-housed mice in the DVC of different mouse models and ongoing projects. The APP23 line was used to reproduce a distinct hyperactive phenotype (Schreyer et al., 2021). APP23 offspring were weaned and genotyped at 3–4 weeks of age and littermates of the same genotype and sex were group-house, predominantly in pairs. APP23 cohorts used for LabMaster or DVC were not identical. LabMaster activity was reevaluated from existing data and DVC activity data collected from a recent project. A second project investigating a metabolic phenotype of KO mice was monitored in the DVC to directly compare activity profiles with LabMaster measurements. LabMaster measurements were performed in the last week of intervention and compared to DVC activity data from the previous week. Furthermore, a type-1-like diabetes model was kept in the DVC to recapitulate its specific hypoactive phenotype (Kou et al., 2014; Ramadan et al., 2006). Female C57Bl/6J mice were injected with Streptozotocin (STZ, 55 mg per kg body weight) or vehicle control (CTR) for five consecutive days to induce pancreatic islet β-cell destruction. 12 CTR (6 cages) and 16 STZ-treated mice (8 cages) were pair-housed in the DVC 2 weeks before and 8 weeks after treatment. All mice were also measured using the LabMaster in week 8 for comparison of activity profiles.

### 2.5 Statistical analysis

Graphs were generated in Tecniplast Analytics (Tecniplast) and GraphPad Prism 9 (GraphPad). Statistical analyses were performed in GraphPad Prism 9. Activity data from DVC are presented as activity index (percentage/h) and from LabMaster as activity counts (counts/h) and movement distance as animal tracking distance (cm/h) and DistD (cm/h), respectively. Graphical representation of data is presented as mean ± SEM. Statistical significance was set as *p ≤ 0.05, **p ≤ 0.01, and ***p ≤ 0.001. Normality distribution of data was analyzed by the Shapiro–Wilk test and equality of variances by Levene’s test. According to data distribution, unpaired two-tailed *t*-test with Welch’s correction was performed to compare the differences between two groups and two-way ANOVA with Bonferroni multiple comparisons test for *post-hoc* analysis for more groups or repeated measures, respectively. To compare the activity data between DVC and LabMaster in different units, *Z*-scores were calculated for every time point of respective measurement (circadian profiles or day and night phase activity) using *Z* = (*x* − *μ*)/*σ*. *Z*-scores were statistically analyzed by (rm) two-way ANOVA with Bonferroni multiple comparisons test for *post hoc* analysis. In addition, the standardized effect size of genotype or treatment (WT vs. KO, WT vs. TG, CTR vs. STZ) analyses was calculated using Hedges’ *g* (*g*, analog to Cohen’s *d*), corrected for small/unequal sample size by *g* = (*μ*_1_ − *μ*_2_)/√(*σ*_1_^2^ + *σ*_2_^2^/2) × (*n*_1_ + *n*_2_ − 3)/(*n*_1_ + *n*_2_ − 2.25) × (√((*n*_1_ + *n*_2_ − 2)/(*n*_1_ + *n*_2_))) for each system and compared to estimate difference detection potential. To further evaluate whether measurements of both systems had similar effect size values, we determined the variance of Hedges’ *g* by var (*g*) = (1 − (3/(4 (*n*_1_ + *n*_2_) − 9)))^2^ × (((*n*_1_ + *n*_2_)/(*n*_1_ × *n*_2_)) + g^2^/(2 (*n*_1_ + *n*_2_))) to estimate a higher practical relevance of one system. These data are listed in the respective tables.

## 3 Results

### 3.1 DVC illustrates locomotor activity index and circadian rhythm of knockout and WT mice

For our phenotyping pipeline, we housed the experimental cohorts of our different mouse models separated by genotype to characterize specific diet and drink intake. So, we came up with the idea to investigate whether DVC’s intra-cage sensor data for activity could provide valid circadian activity profiles. First, we did a rough screening of the cage-based activity index in Analytics (Tecniplast), visualized on a monthly, weekly, and daily basis to present activity metrics (Figures 1A–C). The Animal Locomotion Index (Smoothed) is based on measurements of activation density per cage (Iannello, 2019) and is averaged as daily activity profile, in which basic circadian patterns can be recognized. For further evaluation, we exported the activity data of each cage (per hour) and calculated the individual mouse activity by dividing it by the number of mice in the respective cage. We started by analyzing data from a metabolic project. Here, male WT and KO mice were single- or pair-housed (50%). Single housing was unintentional based on respective litter size or the occurrence of intra-cage aggression (for each diet, 4–6 cages/genotype). In the case of intra-cage aggression, the aggressor mouse was separated and assigned to a new cage. This allowed us to track mouse activity throughout the experiment in a genotype-specific fashion. Before, it was not feasible due to infrastructural limitations, as we only have a busy booked LabMaster device for short-term measurements of indirect calorimetry and activity. Therefore, the LabMaster system is only available for such measurements one time or a maximum of two times during an entire experiment.

We also visualized the DVC activity data of western diet (WD)-fed mice as continuous cage activity during the 3 month intervention (84 days, Figure 1D), as averaged weekly activity (Figure 1E), and as mean daily circadian rhythm (Figure 1F). Apart from the mean daily activity profile, the smoothed weekly and overall activity indices did not provide intuitively clear curves indicating differences. However, the mean circadian profile suggested a potentially lower activity in KO, possibly concealed by averaging over the experiment. From LabMaster analysis, we only analyzed the final week of the dietary interventions, but with the DVC we were able to investigate activity for each week separately. Therefore, our aim was to process the DVC data to derive statistically analyzable and scientifically presentable values.

### 3.2 Processed DVC data for scientific evaluation: visualization of circadian profiles

**Figure 2:**
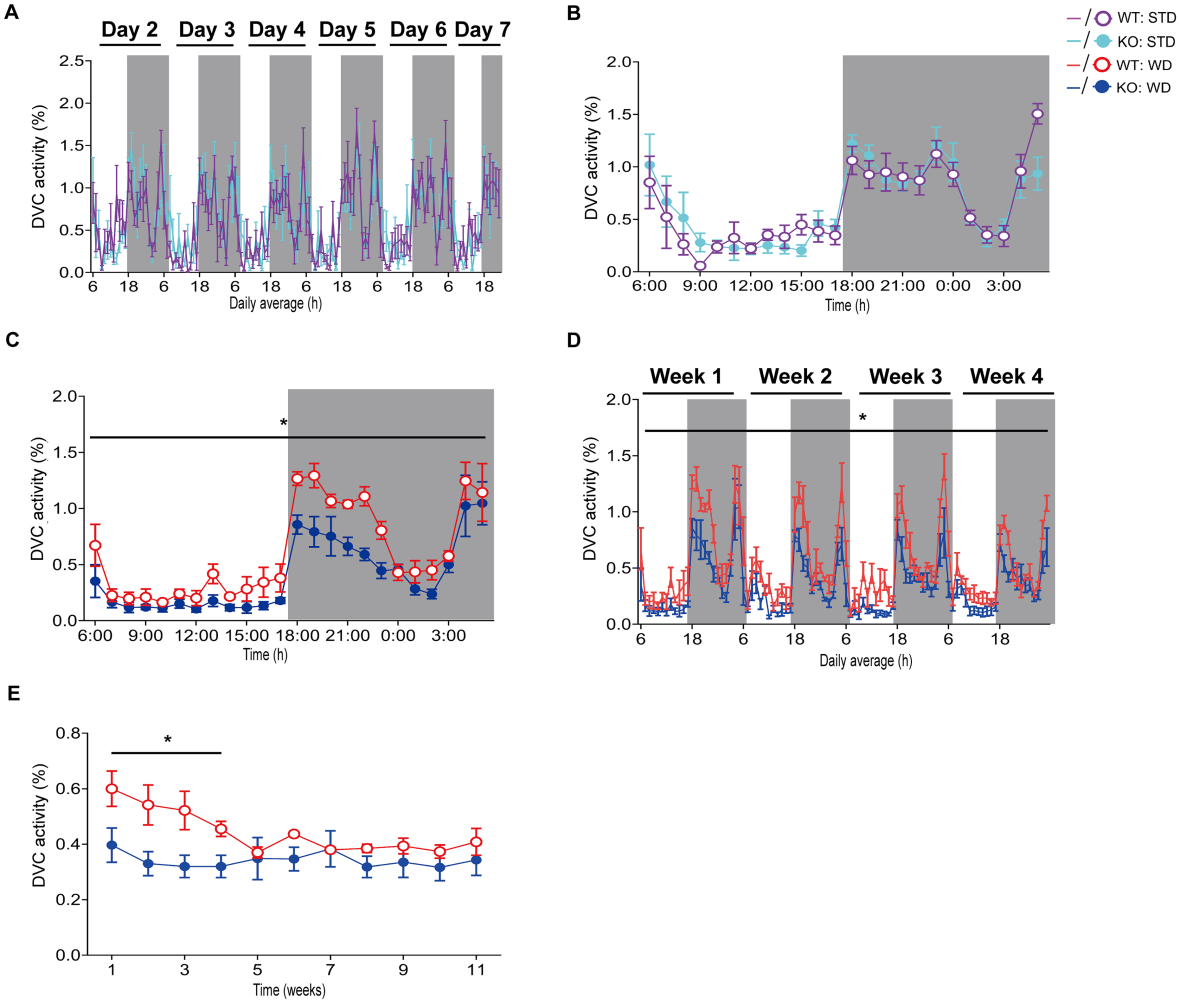
Detailed analysis and scientific presentation of processed DVC activity data of a mouse model on STD or WD. **(A,B)** Activity of WT and KO mice over week 1 **(A)** of STD feeding and the corresponding average daily circadian rhythm **(B)**. **(C)** The average daily activity as circadian rhythm of WT and KO mice on WD over the first week (genotype: *F* (1, 10) = 5.283, *p* = 0.044). **(D)** Weekly activity pattern for the first 4 weeks of WD (genotype: *F* (1, 10) = 6.774, *p* = 0.026). **(E)** Average daily activity per week of WT and KO during the entire WD intervention (week 1: *p* = 0.044, week 2: *p* = 0.035, week 3: *p* = 0.036, week 4: *p* = 0.021). Data are presented as mean ± SEM and were analyzed by repeated measures two-way ANOVA with Bonferroni multiple comparisons test **(A–D)** and unpaired two-tailed *t*-test with Welch’s correction **(E)**. *n*_STD_ = 7 vs. 7, *n*_WD_ = 6 vs. 6 for WT and KO. *p ≤ 0.05. Night phases are gray-shaded. KO, knockout; LM, LabMaster; STD, standard maintenance diet; WD, western diet; WT, wild type.

Hence, we processed the DVC activity data of mice from both dietary interventions for a detailed evaluation as described above to obtain single mouse activity profiles. After exporting and processing the activity index data of WT and KO mice fed STD over 84 days in the DVC, we were able to generate valid activity profiles for graphical representation and for their statistical evaluation. The circadian activity illustrated comparable activity patterns of WT and KO mice during STD (Figures 2A–B), not indicating any difference. During WD, we only obtained similarly uninformative and highly variable activity graphs in Analytics as mentioned above, though the mean daily activity profile implied a potentially reduced circadian rhythm in the KO cages. From the recorded data of the first week of WD feeding, we generated the average daily circadian profile and observed a significant reduction of nocturnal activity in KO mice (Figure 2C). This activity phenotype was robust during the first month of the WD intervention but was only observed there (Figure 2D). Afterwards, the activity of WT mice also decreased due to diet-induced obesity-mediated body weight gain (Figure 2E). Such was also described in a study by Milhem et al. (2024). We would have missed this early KO phenotype because we can generally only perform short-term measurements. Usually, activity and indirect calorimetry of mouse models are assessed before the start and at the end of the specific experiment due to the infrastructural limitations, and not having sufficient monitoring time available in our LabMaster system. The risk of not recognizing specific differences is likely to be an issue for longitudinal studies in particular.

### 3.3 DVC system detected similar activity changes in mice by genotype compared to the calorimetry system under diet intervention

To validate the accuracy, consistency, and scientific quality of our activity profiles derived from DVC monitoring, we directly compared LabMaster measurements of locomotor activity counts of WT and KO mice in the last week of STD or WD intervention with the DVC recordings from the previous week.

**Figure 3:**
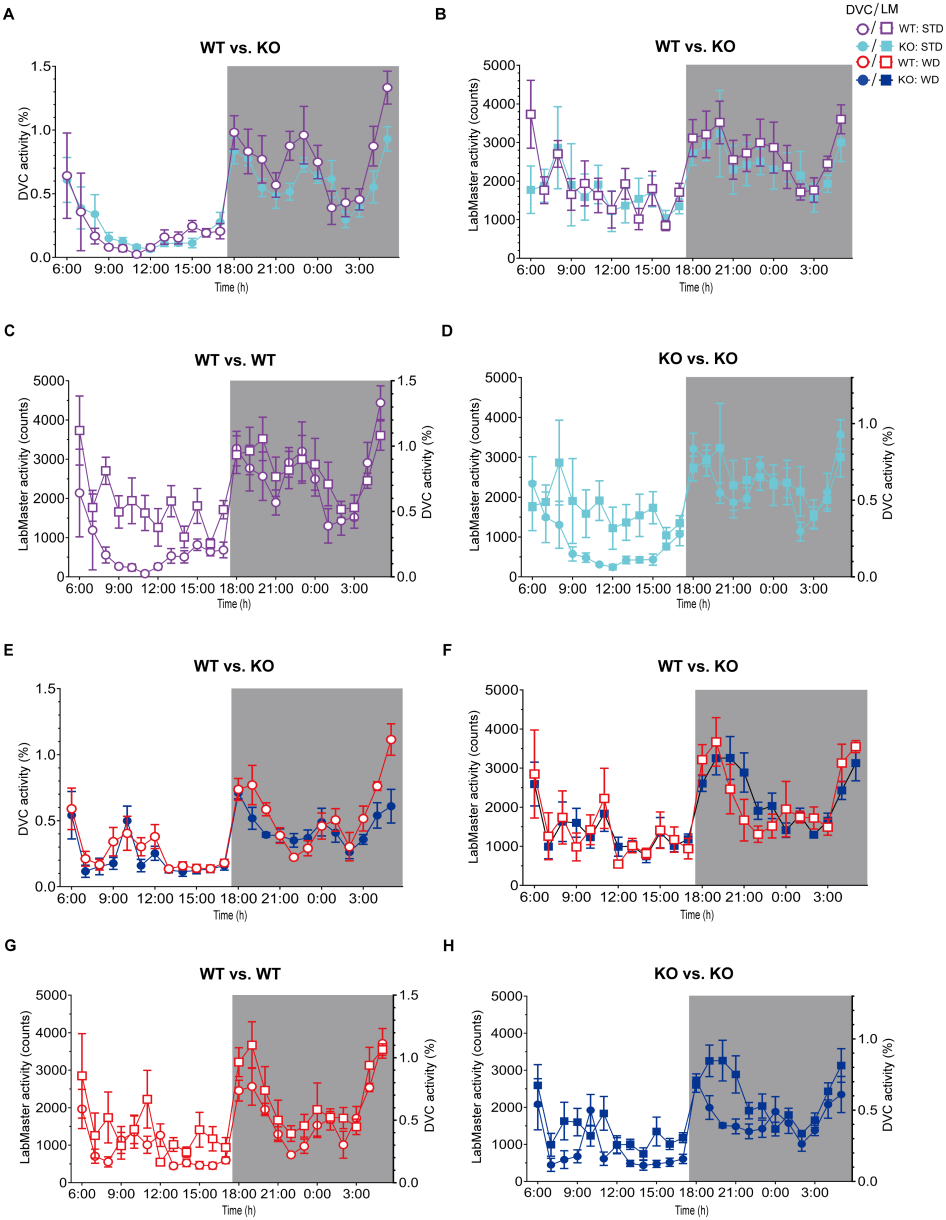
Illustration of processed DVC and LabMaster data for comparison of activity measurements. **(A,B)** Mean daily activity of the same KO and WT mice on STD was monitored via DVC in week 11 **(A)** and measured by LabMaster in week 12 **(B)**. **(C,D)** Overlay of circadian activity curves data from DVC and LabMaster for WT **(C)** or KO **(D)**. **(E,F)** Corresponding activity analyses during WD from DVC in week 11 **(E)** and LabMaster in week 12 **(F)**. **(G,H)** Curve overlays of both systems for WT **(G)** and KO **(H)**. Data are presented as mean ± SEM and were analyzed by repeated measures two-way ANOVA with Bonferroni multiple comparisons test (genotype: **(A)**: *p* = 0.25, **(B)**: *p* = 0.46, **(E)**: *p* = 0.28, **(F)**: *p* = 0.99). *n*_STD_ = 7 vs. 7, *n*_WD_ = 6 vs. 6 for WT and KO. Night phases are gray-shaded. KO, knockout; LM, LabMaster; STD, standard maintenance diet; WD, western diet; WT, wild type.

**Figure 4:**
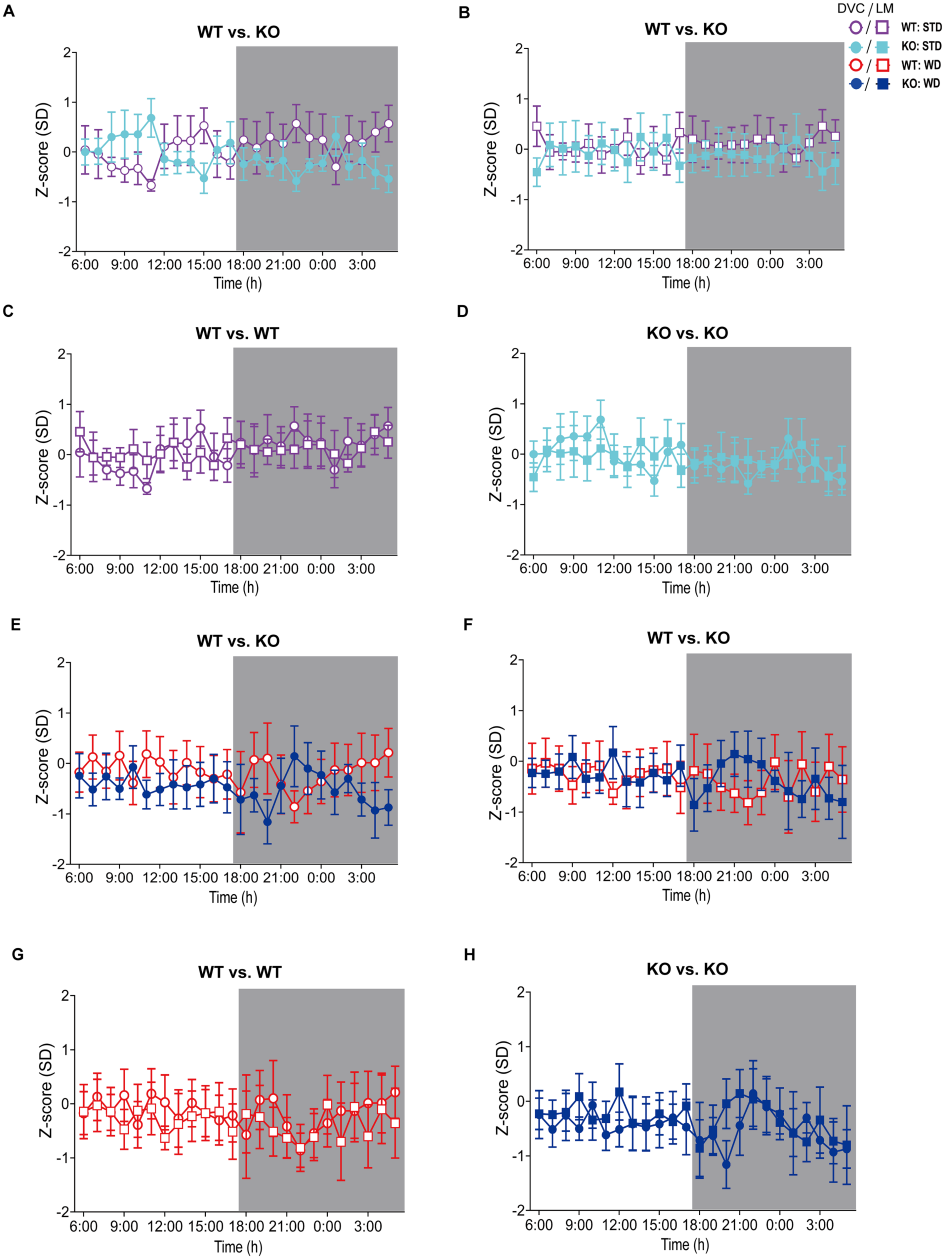
Visualization of *Z*-scores calculated from DVC and LabMaster activity data of WT and KO cohorts. **(A–D)** *Z*-scores of circadian profiles from mice fed STD, comparing data between genotypes **(A,B)** from DVC **(A)** or LabMaster **(B)** as well as intra-genotypic comparisons of DVC and LabMaster **(C,D)** of WT **(C)** and KO **(D)**. **(E–H)** *Z*-score analyses of WD-fed mice, comparing genotypes with each other **(E,F)** from DVC **(E)** or LabMaster data **(F)**, as well as intra-genotypic comparisons of DVC and LabMaster **(G,H)** for WT **(G)** and KO **(H)**. Data are presented as mean ± SEM. Same WT and KO mice on STD (*n* = 7) or WD (*n* = 6) were successively recorded in both systems. Inter- and intra-genotypic comparisons were evaluated by repeated measures two-way ANOVA (genotype: **(A)**: *p* = 0.32, **(B)**: *p* = 0.45; system, **(C)**: *p* = 0.98, **(D)**: *p* > 0.99; genotype, **(E)**: *p* = 0.58, **(F)**: *p* = 0.97; system, **(G)**: *p* = 0.75, **(H)**: *p* = 0.75) with Bonferroni’s multiple comparisons test (**(A–H)**: all adjusted *p* = not significant). Night phases are gray-shaded. KO, knockout; LM, LabMaster; SD, standard deviation; STD, standard maintenance diet; WD, western diet; WT, wild type.

On STD, circadian rhythm from DVC (week 11) showed similar curve progression for both genotypes, in which KO tends to move slightly less during dark phase relative to WT mice, which is not statistically significant (Figure 3A). This was consistent with the LabMaster measurements (week 12), which showed comparable circadian profiles, also with a slightly reduced nocturnal activity of KO (Figure 3B). The more variable curve progression, especially during the day phase, is probably due to the fact that mice were transferred into new cages with fresh bedding and moved to another room for the LabMaster measurements and this stimulated their exploratory behavior. To reduce this, all recorded data are excluded from measurements until the beginning of the first night phase. Such variable data were not observed within the DVC monitoring due to the long-term home cage accommodation, only partial cage change, as well as the exclusion of the regular bedding change day from the weekly activity data analyses. The graphical overlay of the data from both systems revealed similar, matching curves, particularly the pattern during the night phase, for WT (Figure 3C) and KO (Figure 3D), respectively. Similar findings, including the noisy day phase, were observed comparing last weeks of the WD intervention (Figures 3E,F), where the early activity phenotype of KO, as shown in Figure 2E, was no longer obvious. The overlay indicated convincing activity profiles derived from DVC versus LabMaster (Figures 3G,H). In the WD intervention, an even stronger curve-matching of the systems was observed, as obese mice were overall less active, especially during the day phase, which attenuated the home cage effect-dependent difference. However, these graphical comparisons provide no statistical evidence. As both systems measure in different units, a direct comparison is not possible. We, therefore, calculated the *Z*-scores to statistically compare the data from DVC and LabMaster. For WT and KO measurements on STD, we revealed similar *Z*-score outcomes statistically corroborating the consistency of the activity measurements from DVC and LabMaster (Table 1). However, the comparisons of Hedges’ *g* effect size values of WT-KO indicated higher relevance for night phase measurements in the DVC, possibly due to lower variances of values. Beyond that, there are no inter-genotypic differences evident for STD (Figures 4A,B). The intra-genotypic comparisons were not statistically different and showed high concordance of *Z*-scores for the circadian profiles (Figures 4C,D). The *Z*-scores from activity measurements of both systems of WD-fed mice showed similar inter-genotypic tendencies (Figures 4E,F), while the intra-genotypic comparisons were statistically indistinguishable (Table 2 and Figures 4G,H). Furthermore, we determined the effect size in our cohorts using Hedges’ *g* to draw conclusions about how accurately both two systems reflect the degree of effect of activity between the genotypes. The effect size is even higher for DVC monitoring than for the LabMaster measurements for STD and WD cohorts (Tables 1, 2).

**Table 1:** Statistical evaluation of activity profiles of STD-fed WT and KO mice (*n* = 7 per genotype) consecutively measured with DVC and LabMaster.

		*Z*-scores of activity measurements of WT								*Z*-scores of activity measurements of KO								Effect size	
		1	2	3	4	5	6	7	*p*	1	2	3	4	5	6	7	*p*	*g*	var (*g*)
DP	DVC	−0.6	−0.9	−0.6	2.1	0.2	0.2	−0.5	>0.99	−1.0	−1.1	−1.0	1.1	1.1	0.4	0.4	>0.99	0.48	0.251
	LM	−1.0	0.7	−0.5	1.3	−0.1	−1.3	0.9		−0.2	−1.2	−0.5	−0.4	1.9	0.7	−0.1		0.28	0.255
NP	DVC	−0.4	1.6	−0.2	0.8	−1.1	−1.1	0.4	>0.98	−0.1	1.3	−0.7	−1.1	−1.1	0.8	0.8	>0.99	0.56	0.266
	LM	−1.1	−0.1	−1.1	1.7	0.8	−0.1	0.0		1.6	−1.7	0.0	−0.3	0.1	0.6	−0.3		0.28	0.255

*Z*-scores of each measurement were calculated for direct comparison by two-way ANOVA to verify the consistency of measurements of both systems. Hedges’ *g* was determined to quantify the respective effect sizes of each measurement and their variances to estimate the statistical uncertainty of *g* between measurements. DP, day phase; *g*, Hedges’ g effect size; KO, knockout; LM, LabMaster; NP, night phase; *p*, *p*-value of two-way ANOVA comparing *Z*-scores from same genotype; STD, standard diet; var (*g*), variance of *g*; WT, wild type.

**Table 2:** Statistical evaluation of activity profiles of WD-fed WT and KO mice (*n* = 6 per genotype) consecutively measured with DVC and LabMaster.

		*Z*-scores of activity measurements of WT							*Z*-scores of activity measurements of KO							Effect size	
		1	2	3	4	5	6	*p*	1	2	3	4	5	6	*p*	*g*	var (*g*)
DP	DVC	−0.8	−1.1	0.9	0.9	0.9	−0.9	>0.99	−0.7	−0.7	1.3	1.3	−0.4	−0.7	>0.93	0.36	0.287
	LM	−1.1	−0.8	−0.3	1.4	−0.3	1.0		1.4	−0.6	1.0	−1.2	−0.6	0.0		0.25	0.284
NP	DVC	0.5	−1.7	0.6	0.6	0.6	−0.7	>0.95	−0.6	−1.1	1.0	1.0	0.6	−1.0	>0.99	0.68	0.307
	LM	−1.1	−0.6	1.3	1.0	−0.8	0.3		1.6	−0.9	0.5	−1.1	0.3	−0.3		0.52	0.284

*Z*-scores of each measurement were calculated for direct comparison by two-way ANOVA to verify the consistency of measurements of both systems. Hedges’ *g* was determined to quantify the respective effect sizes of each measurement and their variances to estimate the statistical uncertainty of *g* between measurements. DP, day phase; *g*, Hedges’ *g* effect size; KO, knockout; LM, LabMaster; NP, night phase; *p*, *p*-value of two-way ANOVA comparing *Z*-scores from same genotype; TT, two-tailed *t*-tests comparing *g* (WT vs. KO) between DVC and LM; var (*g*), variance of *g*; WD, western diet; WT, wild type.

### 3.4 Comparison of movement distance measurements of both systems

As the activity profiles of both systems can only be indirectly compared, we also evaluated distance of movements. This parameter is recorded in the same unit for both systems. To be able to directly analyze distance between DVC and LabMaster, we exploited data from the same experiments as above but only used data of mice, which had to be single-housed already in the DVC before or during the experiment. Although this was only a small sample, a possible social effect is therefore not relevant here.

**Figure 5:**
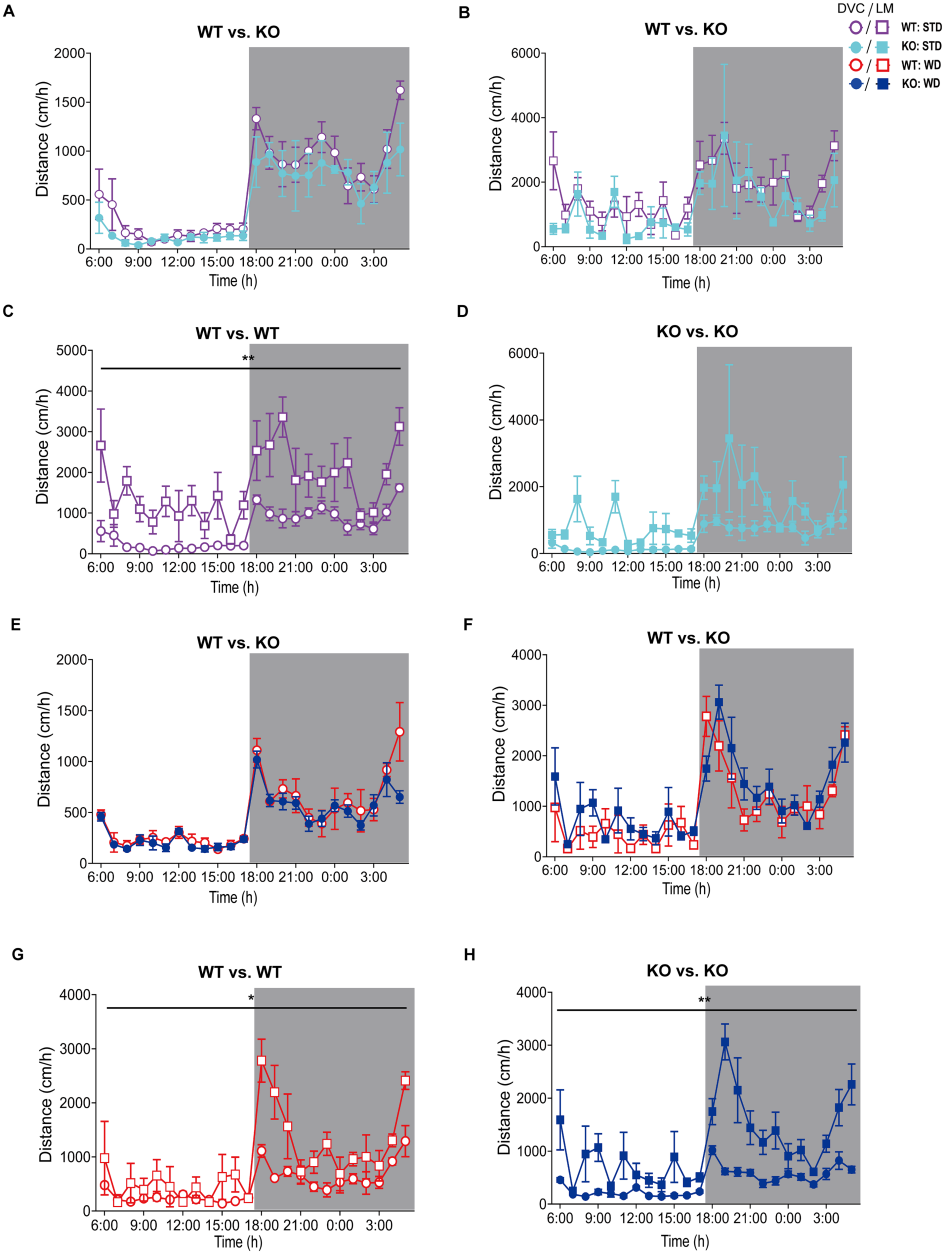
Analysis of movement distance of single-housed WT and KO mice recorded by DVC versus LabMaster. **(A–D)** Mean daily movement distance of STD-fed mice comparing genotypes **(A,B)** from DVC **(A)** or LabMaster **(B)** as well as intra-genotypic comparisons of DVC with LabMaster **(C,D)** of WT **(C)** and KO **(D)**. **(E–H)** Distance analyses of WD-fed mice, comparing genotypes with each other **(E,F)** monitored in the DVC **(E)** or LabMaster **(F)**, as well as intra-genotypic comparisons between DVC and LabMaster **(G,H)** only for WT **(G)** or KO **(H)**. All data are presented as mean ± SEM. Analysis of data from only single-housed WT (*n* = 5) and KO (*n* = 3) during STD **(A–D)** or WT (*n* = 3) and KO mice (*n* = 4) on WD **(E–H)**, which were successively measured in DVC (week 11) and LabMaster (week 12). Inter- and intra-genotypic comparisons were analyzed by repeated measures two-way ANOVA (genotype: **(A)**: *p* = 0.20, **(B)**: *p* = 0.21; system, **(C)**: *p* = 0.003, **(D)**: *p* = 0.09; genotype, **(E)**: *p* = 0.20, **(F)**: *p* = 0.30; system, **(G)**: *p* = 0.03, **(H)**: *p* = 0.001). Night phases are gray-shaded. KO, knockout; LM, LabMaster; STD, standard maintenance diet; WD, western diet; WT, wild type.

We did not observe genotypic differences in distance of WT and KO mice on STD, neither with the DVC (Figure 5A) nor with the LabMaster (Figure 5B). However, these graphs depicted a lower distance but also less measurement variation for the DVC (Table 3). Direct analysis of the inter-genotypic data from both systems showed that WT covered a higher distance during the LabMaster measurement as in the DVC (Figure 5C). This difference was not statistically evident when comparing the two KO measurements (Figure 5D). As in the activity analyses, we also found no genotypic differences of distance during WD in the DVC (Figure 5E) or in the LabMaster measurements (Figure 5F). However, the movement distance covered by WD-fed mice was lower in the LabMaster, which was not observed in the DVC recordings, where the distance between STD and WD was quite similar. Therefore, intra-genomic analyses between DVC and LabMaster measurements revealed reduced distance for WT–WT (Figure 5G) as well as KO–KO comparisons (Figure 5H). The variance of DVC measurements is lower also on WD, which is consistent with a reduced distance (Table 3), but all these distance analyses lack power due to the very small sample size.

**Table 3:** Mean, SD, and *Z*-score analysis of distance data of single-housed WT and KO on STD and WD measured in DVC and LabMaster.

STD		WT		*Z*-scores					KO		*Z*-scores		
		Mean	SD	1	2	3	4	5	Mean	SD	1	2	3
DP	DVC	213	145	−0.08	1.73	−0.39	−0.77	−0.50	120	16	1.12	−0.80	−0.32
	LM	1,209	433	−0.08	−0.70	1.57	0.20	−1.00	707	246	0.84	−1.11	0.27
NP	DVC	983	250	0.43	−0.62	−0.64	1.57	−0.74	799	329	−0.28	−0.83	1.11
	LM	2,111	701	0.82	−0.82	1.27	−0.30	−0.97	1720	977	−0.13	−0.93	1.06

WD		WT		*Z*-scores			KO		*Z*-scores			
		Mean	SD	1	2	3	Mean	SD	1	2	3	4
DP	DVC	237	57	0.92	−1.07	0.15	212	31	−0.52	−0.52	−0.46	1.50
	LM	455	156	−0.32	−0.80	1.12	692	189	0.39	−0.39	1.16	−1.16
NP	DVC	696	70	1.14	−0.73	−0.41	598	86	−0.33	−1.21	1.12	0.42
	LM	1,385	261	−0.29	−0.82	1.11	1,560	314	0.60	−0.59	1.07	−1.08

DP, day phase; KO, knockout; LM, LabMaster; NP, night phase; WD, western diet; WT, wild type.

### 3.5 Transfer and application of the DVC activity profile evaluation to other mouse models

**Figure 6:**
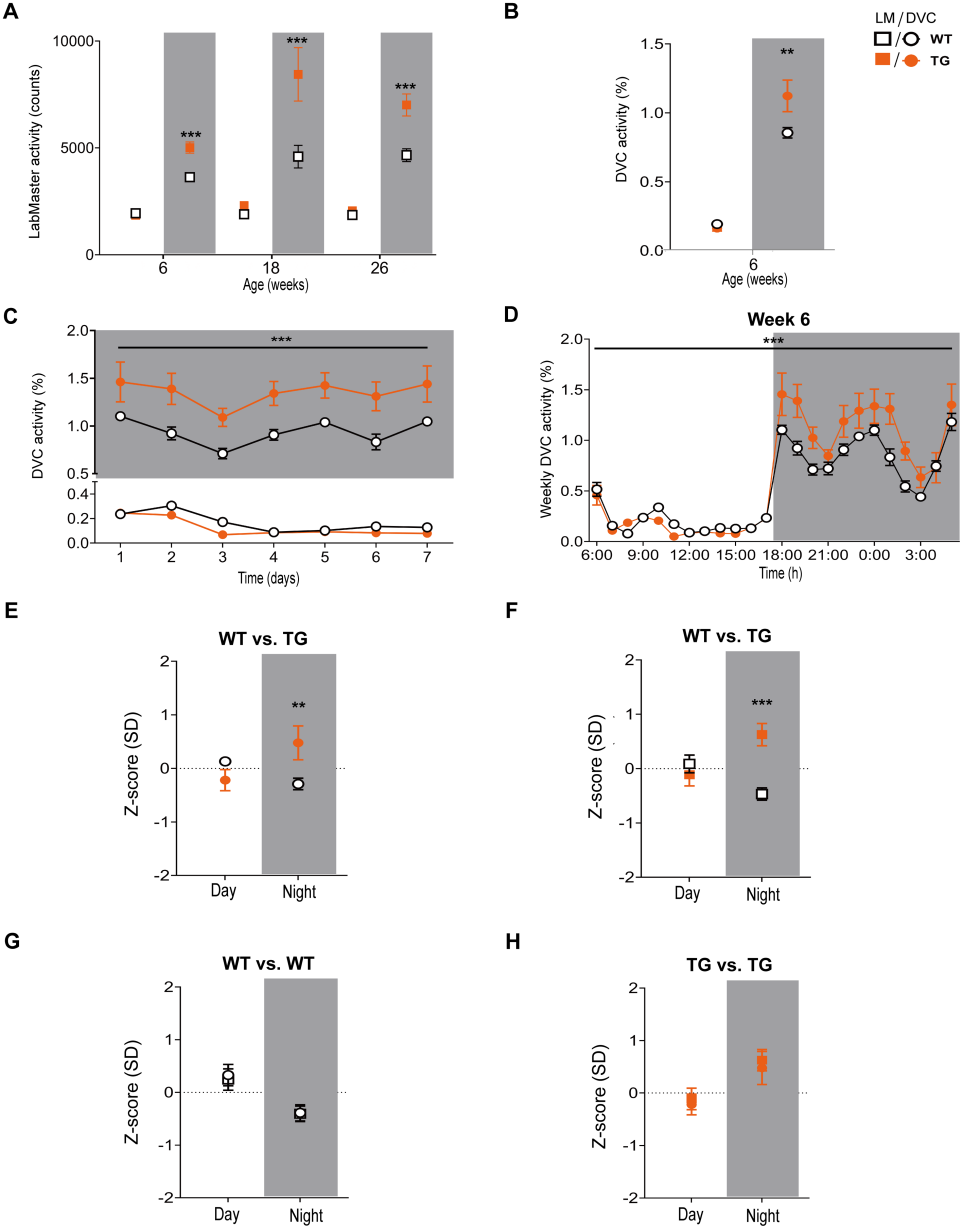
Activity measurements and *Z*-score analysis of young APP23 females with described nocturnal hyperactivity using the DVC. **(A)** Reanalysis of previously recorded day and night phase activity of WT and APP23 transgenic (TG) female mice, single-housed during LabMaster measurement at different ages (genotype: *F* (1, 206) = 52.69, *p* < 0.001, NP, all *p* < 0.001). **(B)** Mean day and night phase activity of 6 week-old group-housed WT and TG females monitored with the DVC (genotype: *F* (1, 44) = 5.319, *p* = 0.025, NP: *p* = 0.025). **(C)** Average day and night activity during week 6 on STD (genotype: *F* (1, 154) = 60.86, *p* < 0.001). **(D)** Circadian activity rhythm of 6 week-old WT and STZ females (genotype: *F* (1, 528) = 31.50, *p* < 0.001). **(E–H)** *Z*-scores of mean day and night activity data comparing WT and TG mice from two different APP23 cohorts **(E,F)** recently monitored using the DVC **(E)** or previously measured with the LabMaster **(F)** and intra-genotypic DVC and LabMaster comparisons **(G,H)** of WT **(G)** and TG **(H)**. All data are presented as mean ± SEM. Independent groups of WT (LabMaster: *n* = 35, DVC: *n* = 15) and TG mice (LabMaster: *n* = 26, DVC: *n* = 9) were separately monitored in one of the two systems. Inter- and intra-genotypic comparisons were evaluated by two-way ANOVA with Bonferroni’s multiple comparisons test (genotype: **(E)**: DP: *p* = 0.28, NP: *p* = 0.004; **(F)**: DP: *p* = 0.82, NP: *p* = 0.009; system; **(G)**: DP: *p* > 0.99, NP: *p* > 0.99; **(H)**: DP: *p* > 0.99, NP: *p* > 0.99). *p ≤ 0.05, **p ≤ 0.01, and ***p ≤ 0.001. Night phases are gray-shaded. DP, day phase; LM, LabMaster; NP, night phase; SD, standard deviation; TG, APP23 transgenic mice; WT, APP23 wild type mice.

Next, we aimed to verify our approach of data processing to generate scientific activity profiles. Therefore, we reinvestigated the transgenic AD model APP23, for which we previously described divergences in activity (Schreyer et al., 2021). We observed that APP23 females exhibit elevated night phase activity during youth adulthood, assessed using the LabMaster (Figure 6A). For this study, we reanalyzed already recorded DVC data of APP23 mice to corroborate the activity phenotype of young APP23 females. With the DVC, increased activity during the night phase was also evident (Figure 6B). We observed an elevated nocturnal activity as early as 6 weeks of age, however, averaging day and night phase led to a loss of day-specific significance (Figure 6C). The circadian activity profile over the first week clearly showed increased activity during the night phase (Figure 6D) in accordance with LabMaster (Figures 6A, 6 weeks). Evaluating measurements of both systems using the *Z*-scores showed accurate conformity replicating the inter-genotypic nocturnal difference of APP23 TG (Figures 6E,F), even with independent measurements from different mice of the same genotypes. Intra-genotypic comparisons were almost identical (Figures 6G,H). No differences in activity were found between measurements of both systems (Table 4). The Hedges’ *g* effect size of both measurements also matched (Table 4). The fact that the effect size is higher for the LabMaster (night phase) is due to the number of mice used (35 and 26 for WT and TG). Although the number of mice (15 WT and 9 TG) analyzed in DVC was significantly lower, a similarly high effect size of the activity measurement could be achieved. Whether this effect is due to the home cage accommodation or superior activity measurements cannot be answered here. Our approach enabled us to precisely validate the findings of the LabMaster activity measurements using the DVC data analysis.

**Table 4:** *Z*-scores, Hedges’ *g*, and variance of *g* from activity data comparing 2 cohorts of WT or APP23 TG mice independently recorded via DVC or LabMaster.

		1	2	3	4	5	6	7	8	9	10	11	12	13	14	15	16	17	18	*p*
*Z*-scores of activity measurements of WT mice																				
DP	DVC	−1.2	0.1	1.9	1.9	0.7	0.7	−0.1	−0.1	0.1	0.1	−0.8	−0.8	−0.6	−0.6	−1.5				>0.98
	LM	−0.1	−1.4	−0.5	−0.2	−0.1	1.1	−0.7	−0.4	−0.1	−1.8	0.1	−0.5	−1.6	1.5	0.3	−0.1	−2.2	0.1	
		0.2	−1.1	1.1	1.4	0.1	−0.9	−0.3	2.3	0.7	−0.1	0.4	−1.3	0.7	0.90	0.33	0.40	1.57		
NP	DVC	−1.7	−1.7	−0.1	−0.1	−0.1	−0.1	0.1	0.1	1.6	1.6	0.9	0.9	−0.9	−0.9	0.3				>0.99
	LM	−0.1	−0.9	0.1	−0.3	0.1	−0.7	0.74	0.7	0.4	−1.2	2.3	0.1	−0.5	1.8	1.3	−0.6	−1.3	0.8	
		−0.2	−0.6	0.7	0.8	0.5	−0.9	−1.1	1.0	−1.0	−0.5	2.6	−0.1	−0.2	0.1	−1.0	−1.3	−0.4		
*Z*-scores of activity measurements of TG mice																				
DP	DVC	2.8	−1.9	−1.8	0.1	−1.5	−1.5	−1.8	−0.7	−0.7										>0.99
	LM	−2.7	−1.1	−0.1	0.1	−1.1	−1.0	−1.2	0.9	−1.4	−0.2	−0.1	−0.6	−1.5	0.4	0.3	−1.1	−0.1	1.5	
		2.2	−0.1	0.8	−0.3	1.3	−0.1	0.7	−1.1											
NP	DVC	4.0	2.6	2.6	6.4	0.4	0.4	−0.6	0.1	0.1										>0.99
	LM	0.3	0.1	0.5	1.1	2.8	3.9	1.4	3.0	4.7	1.8	1.1	0.7	−0.4	2.9	2.2	−1.1	4.5	2.0	
		3.2	2.7	2.7	−0.3	0.9	1.3	0.4	−0.1											

Effect size *g* and its variance of DVC and LM measurements									
		*n* (WT)	*n* (TG)		*g*	var (*g*)		*g*	var (*g*)
	DVC	15	9	DP	0.75	0.066	NP	1.10	0.177
	LM	35	26		0.15	0.168		1.26	0.078

DP, day phase; *g*, Hedges’ *g* effect size; KO, knockout; LM, LabMaster; NP, night phase; *p*, *p*-value of two-way ANOVA comparing *Z*-scores from same genotype; var (*g*), variance of *g*; TG, transgenic APP23; WT, wild type.

**Figure 7:**
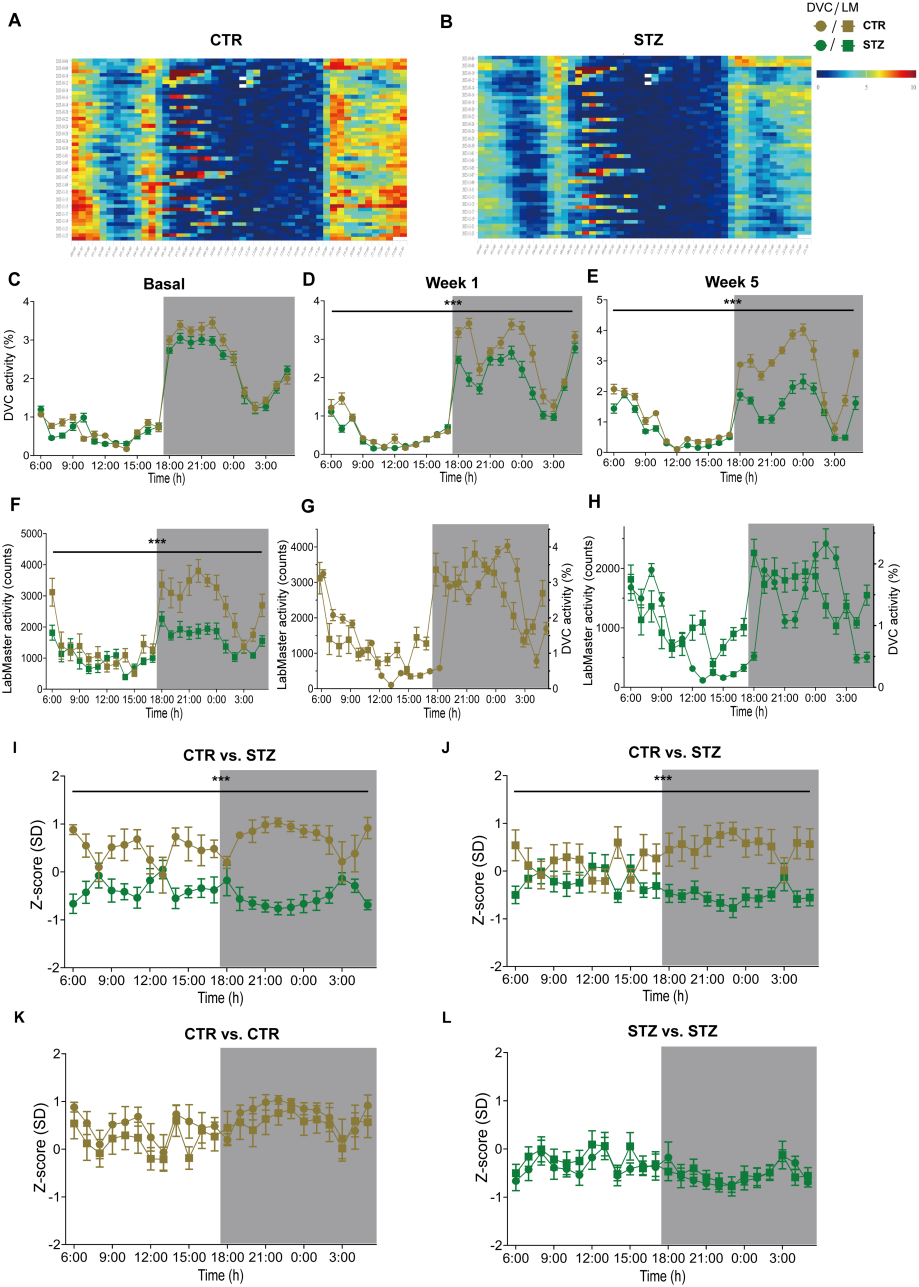
Activity profiles and *Z*-scores analysis of STZ-treated mice from DVC and LabMaster. **(A,B)** Activity heatmaps of control (CTR, **(A)**) and STZ-treated (STZ, **(B)**) pair-housed female mice in 30 min intervals. **(C–E)** Average circadian activity per week before (basal, **(C)**), 1 week (treatment: *F* (1, 26) = 12.19, *p* = 0.002, **(D)**), and 5 weeks (treatment: *F* (1, 26) = 54.96, *p* < 0.001, **(E)**) after treatment assessed using DVC monitoring. **(F)** Circadian activity of CTR and STZ mice was assessed in the final week by LabMaster. **(G,H)** Overlay of activity curves recorded with LabMaster or DVC of female CTR **(G)** and STZ **(H)** mice. **(I–L)** *Z*-scores of circadian activity data from CTR and STZ mice, comparing treatments **(I,J)** recorded in the DVC **(I)** or LabMaster **(J)** or comparing systems **(K,L)** of same CTR **(K)** or STZ mice **(L)**. All data are presented as mean ± SEM. Same CTR (*n* = 12) and STZ mice (*n* = 16) were successively monitored in both systems. Inter- and intra-treatment comparisons were analyzed by repeated measures two-way ANOVA (genotype: **(E)**: *p* < 0.001, **(F)**: *p* < 0.001; system: **(G)**: *p* = 0.13, **(H)**: *p* = 0.15) with Bonferroni’s multiple comparisons test (all adjusted *p* = not significant). *n*_DVC_ = 12 vs. 16, *n*_LM_ = 12 vs. 15 for CTR and STZ. *p ≤ 0.05, **p ≤ 0.01, ***p ≤ 0.001. Night phases are gray-shaded. CTR, vehicle-treated control mice; LM, LabMaster; SD, standard deviation; STZ, streptozotocin-treated mice.

Finally, we evaluated our approach in another mouse model, the Streptozotocin (STZ)-induced pancreatic β-cell destruction type-1-like diabetes model. In this model, a STZ-dependent activity reduction has already been reported (Kou et al., 2014; Ramadan et al., 2006). For this project, mice were pair-housed in the DVC and injected with either STZ or vehicle as controls (CTR). Compared to the CTR group (Figure 7A), the activity heatmap of STZ illustrated that both day and night phase activity are lower after STZ treatment and that this progresses over time (Figure 7B). To evaluate whether these are significant differences, we processed the DVC data as described. In the basal phase before treatment, the activity profiles of CTR and STZ mice were similar (Figure 7C). However, we already observed a decline in night phase activity 1 week after treatment (Figure 7D). This increased considerably until week 5, almost covering the entire night (Figure 7E). The reduced activity of STZ-treated mice was also measured with the LabMaster in the last week (Figure 7F). Overlay of activity profiles from DVC and LabMaster provided comparable curves for CTR or STZ (Figures 7G,H). *Z*-score comparisons revealed a high level of agreement (Table 5). *Z*-scores also reproduced the differences between treatments (Figures 7I,J) and were highly superimposable when comparing the same treatment group from both systems (Figures 7K,L). No significant differences were found between both systems. In this analysis, the same mice were only time-shifted and measured in both systems, but the effect size indicated a higher practical use for the DVC activity data (Table 5). Thus, we were able to corroborate our approach to evaluating activity profiles from the DVC home cage monitoring with minor constraints regarding lower activity due to the social home cage effect.

**Table 5:** *Z*-scores and Hedges’ *g* comparisons of CTR- and STZ-treated mice successively monitored in DVC and LabMaster.

*Z*-scores of activity measurements of CTR mice														
		1	2	3	4	5	6	7	8	9	10	11	12	*p*
DP	DVC	−0.1	−0.1	0.2	0.2	1.6	1.6	−1.0	−1.0	0.6	0.6	−1.3	−1.3	0.48
	LM	−1.3	0.9	−0.1	−0.2	0.2	−0.2	0.2	−1.4	1.0	1.2	−1.5	1.2	
NP	DVC	1.0	1.0	−1.0	−1.0	1.3	1.3	−1.1	−1.1	−0.7	−0.7	0.4	0.4	>0.99
	LM	−0.6	−0.4	0.1	0.5	−0.2	−0.4	−0.4	−0.8	1.2	−0.2	−1.3	2.4	

*Z*-scores of activity measurements of STZ mice																		
		1	2	3	4	5	6	7	8	9	10	11	12	13	14	15	16	*p*
DP	DVC	−2.4	−2.4	−2.5	−2.5	−3.5	−3.5	−2.1	−2.1	−4.3	−4.3	−3.3	−3.3	0.4	0.4	−1.8	−1.8	0.66
	LM	1.0	−1.4	−1.0	0.3	−0.1	0.6	−1.0	−0.2	−0.4	−1.1	−1.8	−1.9	−2.6	−1.3	−1.6	1.0	
NP	DVC	−3.4	−3.4	−2.7	−2.7	−4.9	−4.9	−1.9	−1.9	−5.1	−5.1	−3.9	−3.9	−2.2	−2.2	−2.1	−2.1	>0.98
	LM	−0.5	−1.5	−2.2	−1.1	−1.3	−1.1	−1.9	−0.5	−2.1	−2.1	−2.2	−1.5	−3.0	−2.2	−1.9	−0.5	

Effect size *g* and its variance of DVC and LM measurements									
		*n* (WT)	*n* (TG)		*g*	var (*g*)		*g*	var (*g*)
	DVC	12	16	DP	1.24	0.164	NP	2.09	0.211
	LM	12	15		0.32	0.143		1.08	0.161

CTR, vehicle-treated control group; DP, day phase; *g*, Hedges’ *g* effect size; KO, knockout; LM, LabMaster; NP, night phase; *p*, *p*-value of two-way ANOVA comparing *Z*-scores from same genotype; STZ, streptozotocin-treated group; var (*g*), variance of *g*.

In summary, we could use the DVC activity data for scientific evaluation in a comparable manner to an established systems for activity assessment. Processing the activity data of a pair- or group-house (up to 3) mice of the same genotype or treatment provided valid data for statistical analysis and proper visualization.

## 4 Discussion

Since energy metabolism is of fundamental importance for metabolic research, the accurate and efficient measurement of locomotor activity of laboratory animals is essential. Although previous reports have emphasized the working principles and protocols of DVC monitoring in various (disease) models, its utility for research in multiple fields was poorly investigated, and validation of scientifically robust activity profiles warranted further investigation. The DVC measures activity automatically as a parameter in real time 24/7. Thus, we propose an approach to process the DVC monitoring data to assess individual activity profiles during social housing in the home cage. In our study, we show DVC-derived activity profiles and circadian rhythms from different mouse models. We compared these activity measurements with those of a conventional, established system, the TSE LabMaster, to ensure the consistency of the derived activity profiles. Moreover, we used the DVC in two other mouse models to reproduce their known activity phenotypes to further validate our approach. With it, we obtained longitudinal profiles for daily, weekly, and monthly activity that showed robust and valid circadian rhythms. The locomotor activity (of mice) varies across the lifespan (Brust et al., 2015) and age-related changes in a wide range of behaviors in mice have been demonstrated (Shoji and Miyakawa, 2019). Hence, we propose the DVC as an efficient and 3R-suitable system to assess activity in longitudinal studies, as it combines non-invasive recording of convincing activity profiles with group housing in a home cage.

Systems that measure activity as a parameter, among other things, inevitably share similarities, but often completely differ in the technologies used. The LabMaster from the PhenoMaster family uses an infrared light beam frame (ActiMot) to assess spontaneous locomotor activity (Edelsbrunner et al., 2009). In contrast, the DVC detects intra-cage activity by measuring changes in electromagnetic fields (Iannello, 2019). This is safe for the animals and does not affect them (Burman et al., 2018; Pernold et al., 2019; Recordati et al., 2019). These data are automatically analyzed in a cloud-based platform to support cage maintenance and welfare aspects and also can detect deviations from the “natural” behavior of the preceding days to send alerts. A major advantage of this monitoring is that it is recorded in the home cage without any additional disturbance to the mice, as cage changes and handling affect behavior, circadian clock, and sleeping parameters and cause stress for mice (Balcombe et al., 2004; Rosenbaum et al., 2009; Febinger et al., 2014). Moreover, cage changes are known to influence activity in different strains increasing basal activity over 5 h (Fuochi et al., 2021). Mice are nocturnal and mainly reveal behavioral traits, especially at night (Eckel-Mahan and Sassone-Corsi, 2015), when staff and scientists are not present. Therefore, continuous evaluation of activity profiles and their specific changes, particularly during the night phase, can benefit numerous research areas and pharmacology. As we show here, all our evaluated mouse models exhibited their activity differences in the night phase, which either were identified by specific experimental questions or might otherwise have gone unnoticed. This could be a major confounder of specialized systems that are often only used for short-term monitoring due to infrastructural shortcomings. In addition to these functions, we investigated whether the recorded activity data could also be processed to pass a scientific evaluation and statistical analysis.

Since activity is monitored in different units by DVC and LabMaster, measurements of both systems cannot be statistically analyzed. Therefore, we calculated *Z*-scores to standardize both measurements for a direct comparison. The normalized parameters not only confirm the difference we already observed between genotypes and treatment, respectively, but also revealed almost identical activity measurements of the same genotype/treatment from DVC and LabMaster. All *Z*-score analyses were in statistical agreement. Furthermore, we evaluated effect size using Hedges’ g adjusted for small/unequal sample size for inter-genotypic/treatment comparisons to check the effectivity of both monitoring systems. The effect size of the activity measurements with the DVC system was at least similar, often higher than those from LabMaster indicating a more accurate activity monitoring presumably due to husbandry conditions such as the home cage effect and the social housing.

In our study, we evaluated the DVC activity measurements of a respective mouse model comparing diet-induced obesity-dependent reduction of activity, previously assessed with other conventional systems (Brachs et al., 2018; Greenman et al., 2013; Clemmensen et al., 2015). Consistently, we observed similar activity findings analyzing data of both systems for dietary interventions. We also revealed early differences that we would otherwise have missed due to infrastructural restrictions of our experimental pipeline. Our results show that the DVC can produce continuous, accurate, and comprehensive mouse circadian rhythms in the home cages, preconditioned mice are separated by genotype, treatment, or such and not mixed within that cage. In contrast, other systems often require, e.g., single housing, environment/cage change, sensor implantation, or else affecting welfare, behavior, and ultimately also activity through introduction of external stress. To further validate our approach, we reanalyzed the transgenic AD line APP23 mice, in which we described an elevated night phase activity using the LabMaster system (Schreyer et al., 2021). We corroborated the same hyperactive phenotype by continuous DVC monitoring in group-house female APP23. In addition, we confirmed an STZ treatment-dependent reduction in activity (Kou et al., 2014; Ramadan et al., 2006) using the DVC and specified its occurrence, particularly in the night phase. If assessment of activity/circadian rhythms is required as a single parameter, the continuous home cage data provide consistency and reproducibility while saving effort and reducing stress. However, unlike most other available systems, the DVC can only monitor true cage activity, moisture, and their changes, and does not record real activity profiles of individual mice from each home cage.

Our activity profiles derived from DVC monitoring revealed lower and unnoisier day phase activity compared to the LabMaster measurements indicating an advantage of home cage monitoring of group-housed mice, which do not explore an unfamiliar, new environment resulting in a more sedentary resting phase. The increased noise in the curve progression of LabMaster, particularly during the daytime, is likely due to the mice being transferred to new cages with fresh bedding and moved to another room for the LabMaster measurements. This likely stimulates their exploratory behavior. This elevated activity was not observed throughout our DVC monitoring potentially due to long-term and home cages accommodation, together with exclusion of the regular bedding change day from the weekly activity data analyses. Therefore, we assume that the DVC monitoring provides more “natural” circadian rhythms regarding activity measurements compared to the experimental systems which are mostly used for short-term analyses. Overall, in terms of activity profiles, the DVC seems to better reflect the regular routine of nocturnal animals.

However, a restriction of the DVC monitoring is the inability to measure true individual data for specific mice in the group housing setup. All our evaluations are only possible as mice are separated by genotype, treatment, or intervention. Another DVC limitation is its technology which currently monitors only changes in electromagnetic fields in the cage and what can be derived from these values such as activity, Regularity Disruption Index (Golini et al., 2020), bedding moisture, and leaking bottles. Other systems, such as the TSE LabMaster, primarily analyze several other parameters, such as gas calorimetry (O_2_ and CO_2_) to calculate energy expenditure of single-housed mice, where activity represents only a secondary factor.

The movement distance is measured by both systems in the same unit and should be directly comparable; however, we found that single-housed mice cover less distance in the DVC than in the LabMaster. An explanation could be that an increased exploration behavior of mice transferred to the LabMaster has still more effect on activity and distance of the second day phase and the night phases due to the cage and environment changes as the partial cage change in the DVC. The difference in distance measurements of the two systems might also be related to technical specifications. The LabMaster detects activity and distance with a unique 5 mm sensor spacing. While the DVC monitors activity and distance via a sensor board with 12 electrodes sensing electrical changes once every 0.25 s. Both parameters, activity and distance, are not congruent between these systems, but our relative comparisons between genotypes or treatments show that we are able to detect the same phenotypes using either system.

In conclusion, we evaluated DVC’s monitoring data regarding scientific utilization of circadian activity patterns, their reproducibility as well as comparability to an established, conventional system to ensure valid and robust results. Therefore, we summarized previous observations from different disciplines to corroborate the accuracy of activity profiles. Our approach can be deployed in numerous research areas to assess locomotor activity in a less intrusive and more objective way. In terms of 3R principles, the home cage-based monitoring simultaneously refines animal welfare through social housing, less handling, and finally reduction of stress. Furthermore, high-throughput longitudinal studies can be easily implemented as a DVC rack can accommodate 70 cages, monitoring them 24/7 while additionally assisting with animal care and maintenance.

## Data Availability

The raw data supporting the conclusions of this article will be made available by the authors, without undue reservation.

## References

[ref1] Alonso-Frech F. Sanahuja J. J. Rodriguez A. M. (2011). Exercise and physical therapy in early management of Parkinson disease. Neurologist 17, S47–S53. doi: 10.1097/NRL.0b013e31823968ec 22045326

[ref2] Balcombe J. P. Barnard N. D. Sandusky C. (2004). Laboratory routines cause animal stress. Contemp. Top. Lab. Anim. Sci. 43, 42–51. 15669134

[ref3] Brachs S. Haas W. (2008). Swimming behaviour of *Schistosoma mansoni* cercariae: responses to irradiance changes and skin attractants. Parasitol. Res. 102, 685–690. doi: 10.1007/s00436-007-0812-4 18157546

[ref4] Brachs S. Polack J. Brachs M. Jahn-Hofmann K. Elvert R. Pfenninger A.. (2018). Genetic nicotinamide N-methyltransferase (Nnmt) deficiency in male mice improves insulin sensitivity in diet-induced obesity but does not affect glucose tolerance. Diabetes 68, 527–542. doi: 10.2337/db18-0780, PMID: 30552109

[ref5] Brust V. Schindler P. M. Lewejohann L. (2015). Lifetime development of behavioural phenotype in the house mouse (*Mus musculus*). Front. Zool. 12:s17. doi: 10.1186/1742-9994-12-S1-S17 26816516 PMC4722345

[ref6] Burman O. Marsella G. Di Clemente A. Cervo L. (2018). The effect of exposure to low frequency electromagnetic fields (EMF) as an integral part of the housing system on anxiety-related behaviour, cognition and welfare in two strains of laboratory mouse. PLoS One 13:e0197054. doi: 10.1371/journal.pone.0197054 29771983 PMC5957419

[ref7] Caggiano V. Leiras R. Goni-Erro H. Masini D. Bellardita C. Bouvier J.. (2018). Midbrain circuits that set locomotor speed and gait selection. Nature 553, 455–460. doi: 10.1038/nature25448, PMID: 29342142 PMC5937258

[ref8] Carlson D. J. Dieberg G. Hess N. C. Millar P. J. Smart N. A. (2014). Isometric exercise training for blood pressure management: a systematic review and meta-analysis. Mayo Clin. Proc. 89, 327–334. doi: 10.1016/j.mayocp.2013.10.030 24582191

[ref9] Chew N. W. S. Ng C. H. Tan D. J. H. Kong G. Lin C. Chin Y. H.. (2023). The global burden of metabolic disease: data from 2000 to 2019. Cell Metab. 35, 414–28.e3. doi: 10.1016/j.cmet.2023.02.003, PMID: 36889281

[ref10] Clemmensen C. Finan B. Fischer K. Tom R. Z. Legutko B. Sehrer L.. (2015). Dual melanocortin-4 receptor and GLP-1 receptor agonism amplifies metabolic benefits in diet-induced obese mice. EMBO Mol. Med. 7, 288–298. doi: 10.15252/emmm.201404508, PMID: 25652173 PMC4364946

[ref11] Collaboration NCDRF (2017). Worldwide trends in body-mass index, underweight, overweight, and obesity from 1975 to 2016: a pooled analysis of 2416 population-based measurement studies in 128.9 million children, adolescents, and adults. Lancet 390, 2627–2642. doi: 10.1016/S0140-6736(17)32129-3 29029897 PMC5735219

[ref12] Dakup P. P. Porter K. I. Little A. A. Gajula R. P. Zhang H. Skornyakov E.. (2018). The circadian clock regulates cisplatin-induced toxicity and tumor regression in melanoma mouse and human models. Oncotarget 9, 14524–14538. doi: 10.18632/oncotarget.24539, PMID: 29581861 PMC5865687

[ref13] Eckel-Mahan K. Sassone-Corsi P. (2015). Phenotyping circadian rhythms in mice. Curr. Protoc. Mouse Biol. 5, 271–281. doi: 10.1002/9780470942390.mo140229, PMID: 26331760 PMC4732881

[ref14] Edelsbrunner M. E. Painsipp E. Herzog H. Holzer P. (2009). Evidence from knockout mice for distinct implications of neuropeptide-Y Y2 and Y4 receptors in the circadian control of locomotion, exploration, water and food intake. Neuropeptides 43, 491–497. doi: 10.1016/j.npep.2009.08.007, PMID: 19781771 PMC4359899

[ref15] Fayyaz M. Jaffery S. S. Anwer F. Zil E. A. A. Anjum I. (2018). The effect of physical activity in Parkinson’s disease: a Mini-review. Cureus. 10:e2995. doi: 10.7759/cureus.2995 30245949 PMC6143369

[ref16] Febinger H. Y. George A. Priestley J. Toth L. A. Opp M. R. (2014). Effects of housing condition and cage change on characteristics of sleep in mice. J. Am. Assoc. Lab. Anim. Sci. 53, 29–37. 24411777 PMC3894645

[ref17] Frenz U. Aust L. Noack R. (1995). Calculating locomotor activity and energy utilisation factors from indirect calorimetric measurements. Thermochim. Acta 251, 271–281. doi: 10.1016/0040-6031(94)02060-2

[ref18] Fuochi S. Rigamonti M. Iannello F. Raspa M. Scavizzi F. de Girolamo P.. (2021). Phenotyping spontaneous locomotor activity in inbred and outbred mouse strains by using digital ventilated cages. Lab Anim. 50, 215–223. doi: 10.1038/s41684-021-00793-0 34155410

[ref19] Golini E. Rigamonti M. Iannello F. De Rosa C. Scavizzi F. Raspa M.. (2020). A non-invasive digital biomarker for the detection of rest disturbances in the SOD1G93A mouse model of ALS. Front. Neurosci. 14:896. doi: 10.3389/fnins.2020.00896, PMID: 32982678 PMC7490341

[ref20] Greenman Y. Kuperman Y. Drori Y. Asa S. L. Navon I. Forkosh O.. (2013). Postnatal ablation of POMC neurons induces an obese phenotype characterized by decreased food intake and enhanced anxiety-like behavior. Mol. Endocrinol. 27, 1091–1102. doi: 10.1210/me.2012-1344, PMID: 23676213 PMC5415244

[ref21] Guimarães T. T. Gomes S. M. R. Albuquerque R. Lima A. K. C. Braga G. F. Souza J. B.. (2021). Chronic aerobic training at different volumes in the modulation of macrophage function and in vivo infection of BALB/c mice by Leishmania major. Front. Microbiol. 12:734355. doi: 10.3389/fmicb.2021.734355, PMID: 34616386 PMC8489854

[ref22] Hallal P. C. Victora C. G. Azevedo M. R. Wells J. C. (2006). Adolescent physical activity and health: a systematic review. Sports Med. 36, 1019–1030. doi: 10.2165/00007256-200636120-00003 17123326

[ref23] Handelsman Y. Butler J. Bakris G. L. DeFronzo R. A. Fonarow G. C. Green J. B.. (2023). Early intervention and intensive management of patients with diabetes, cardiorenal, and metabolic diseases. J. Diabetes Complicat. 37:108389. doi: 10.1016/j.jdiacomp.2022.108389, PMID: 36669322

[ref24] Hillar C. Onnis G. Rhea D. Tecott L. (2018). Active state Organization of Spontaneous Behavioral Patterns. Sci. Rep. 8:1064. doi: 10.1038/s41598-017-18276-z 29348406 PMC5773533

[ref25] Hughes A. T. L. Piggins H. D. (2012). Feedback actions of locomotor activity to the circadian clock. Prog. Brain Res. 199, 305–336. doi: 10.1016/B978-0-444-59427-3.00018-6 22877673

[ref26] Iannello F. (2019). Non-intrusive high throughput automated data collection from the home cage. Heliyon 5:e01454. doi: 10.1016/j.heliyon.2019.e01454, PMID: 30997429 PMC6451168

[ref27] Ingebretson J. J. Masino M. A. (2013). Quantification of locomotor activity in larval zebrafish: considerations for the design of high-throughput behavioral studies. Front Neural Circuits. 7:109. doi: 10.3389/fncir.2013.00109 23772207 PMC3677137

[ref28] Jehan S. Zizi F. Pandi-Perumal S. R. McFarlane S. I. Jean-Louis G. Myers A. K. (2020). Energy imbalance: obesity, associated comorbidities, prevention, management and public health implications. Adv Obes Weight Manag Control. 10, 146–161. 33305001 PMC7725222

[ref29] Kamei J. Saitoh A. Iwamoto Y. Funada M. Suzuki T. Misawa M.. (1994). Effects of diabetes on spontaneous locomotor activity in mice. Neurosci. Lett. 178, 69–72. doi: 10.1016/0304-3940(94)90292-5 7816344

[ref30] Kelley A. E. (1993). Locomotor activity and exploration. Techniques in the behavioral and neural sciences, vol. 10: Elsevier, 499–518.

[ref31] Kemoun G. Thibaud M. Roumagne N. Carette P. Albinet C. Toussaint L.. (2010). Effects of a physical training programme on cognitive function and walking efficiency in elderly persons with dementia. Dement. Geriatr. Cogn. Disord. 29, 109–114. doi: 10.1159/000272435, PMID: 20150731

[ref32] Klein C. Budiman T. Homberg J. R. Verma D. Keijer J. van Schothorst E. M. (2022). Measuring locomotor activity and behavioral aspects of rodents living in the home-cage. Front. Behav. Neurosci. 16:877323. doi: 10.3389/fnbeh.2022.877323 35464142 PMC9021872

[ref33] Kodama S. Tanaka S. Saito K. Shu M. Sone Y. Onitake F.. (2007). Effect of aerobic exercise training on serum levels of high-density lipoprotein cholesterol: a meta-analysis. Arch. Intern. Med. 167, 999–1008. doi: 10.1001/archinte.167.10.999 17533202

[ref34] Kopaczka M. Tillmann D. Ernst L. Schock J. Tolba R. Merhof D. (2019). Assessment of laboratory mouse activity in video recordings using deep learning methods. Annu. Int. Conf. IEEE Eng. Med. Biol. Soc. 2019, 3673–3676. doi: 10.1109/EMBC.2019.8857807 31946673

[ref35] Kou Z. Z. Li C. Y. Hu J. C. Yin J. B. Zhang D. L. Liao Y. H.. (2014). Alterations in the neural circuits from peripheral afferents to the spinal cord: possible implications for diabetic polyneuropathy in streptozotocin-induced type 1 diabetic rats. Front. Neural. Circuits. 8:6. doi: 10.3389/fncir.2014.00006 24523675 PMC3905201

[ref36] Latimer-Cheung A. E. Pilutti L. A. Hicks A. L. Martin Ginis K. A. Fenuta A. M. MacKibbon K. A.. (2013). Effects of exercise training on fitness, mobility, fatigue, and health-related quality of life among adults with multiple sclerosis: a systematic review to inform guideline development. Arch. Phys. Med. Rehabil. 94, 1800–28.e3. doi: 10.1016/j.apmr.2013.04.020, PMID: 23669008

[ref37] Mac Phail R. C. Peele D. B. Crofton K. M. (1989). Motor activity and screening for neurotoxicity. J. Am. Coll. Toxicol. 8, 117–125. doi: 10.3109/10915818909009098

[ref38] Makaras T. Montvydiene D. Kazlauskiene N. Stankeviciute M. Raudonyte-Svirbutaviciene E. (2020). Juvenile fish responses to sublethal leachate concentrations: comparison of sensitivity of different behavioral endpoints. Environ. Sci. Pollut. Res. Int. 27, 4876–4890. doi: 10.1007/s11356-019-07211-6 31845261

[ref40] Milhem F. Skates E. Wilson M. Komarnytsky S. (2024). Obesity-resistant mice on a high-fat diet display a distinct phenotype linked to enhanced lipid metabolism. Nutrients 16:10171. doi: 10.3390/nu16010171, PMID: 38202000 PMC10780630

[ref41] Moser V. C. Liu Z. Schlosser C. Spanogle T. L. Chandrasekaran A. McDaniel K. L. (2016). Locomotor activity and tissue levels following acute administration of lambda- and gamma-cyhalothrin in rats. Toxicol. Appl. Pharmacol. 313, 97–103. doi: 10.1016/j.taap.2016.10.020, PMID: 27794438

[ref42] Niederberger E. Parnham M. J. (2021). The impact of diet and exercise on drug responses. Int. J. Mol. Sci. 22:147692. doi: 10.3390/ijms22147692, PMID: 34299312 PMC8304791

[ref43] Nikodijevic O. Jacobson K. A. Daly J. W. (1993). Locomotor activity in mice during chronic treatment with caffeine and withdrawal. Pharmacol. Biochem. Behav. 44, 199–216. doi: 10.1016/0091-3057(93)90299-9 7679219 PMC3557839

[ref44] Pernold K. Iannello F. Low B. E. Rigamonti M. Rosati G. Scavizzi F.. (2019). Towards large scale automated cage monitoring - diurnal rhythm and impact of interventions on in-cage activity of C57BL/6J mice recorded 24/7 with a non-disrupting capacitive-based technique. PLoS One 14:e0211063. doi: 10.1371/journal.pone.0211063, PMID: 30716111 PMC6361443

[ref45] Perse M. (2021). Cisplatin mouse models: treatment, toxicity and translatability. Biomedicines 9:101406. doi: 10.3390/biomedicines9101406, PMID: 34680523 PMC8533586

[ref46] Ramadan W. Dewasmes G. Petitjean M. Loos N. Delanaud S. Geloen A.. (2006). Spontaneous motor activity in fat-fed, streptozotocin-treated rats: a nonobese model of type 2 diabetes. Physiol. Behav. 87, 765–772. doi: 10.1016/j.physbeh.2006.01.012, PMID: 16516253

[ref47] Recordati C. De Maglie M. Marsella G. Milite G. Rigamonti A. Paltrinieri S.. (2019). Long-term study on the effects of housing C57BL/6NCrl mice in cages equipped with wireless technology generating extremely Low-intensity electromagnetic fields. Toxicol. Pathol. 47, 598–611. doi: 10.1177/0192623319852353, PMID: 31117895

[ref48] Reyes-Molina D. Alonso-Cabrera J. Nazar G. Parra-Rizo M. A. Zapata-Lamana R. Sanhueza-Campos C.. (2022). Association between the physical activity behavioral profile and sedentary time with subjective well-being and mental health in Chilean university students during the COVID-19 pandemic. Int. J. Environ. Res. Public Health 19:42107. doi: 10.3390/ijerph19042107, PMID: 35206294 PMC8872099

[ref49] Rosenbaum M. D. VandeWoude S. Johnson T. E. (2009). Effects of cage-change frequency and bedding volume on mice and their microenvironment. J. Am. Assoc. Lab. Anim. Sci. 48, 763–773, PMID: 19930825 PMC2786931

[ref39] Russell W. M. S. Burch R. L. (1960). The principles of humane experimental technique. Med. J. Aust. 1:500. doi: 10.5694/j.1326-5377.1960.tb73127.x

[ref50] Salem G. Cope N. Garmendia M. Pu A. Somenhalli A. Krynitsky J.. (2024). MouseVUER: video based open-source system for laboratory mouse home-cage monitoring. Sci. Rep. 14:2662. doi: 10.1038/s41598-024-52788-9, PMID: 38302573 PMC10834510

[ref51] Schreyer S. Berndt N. Eckstein J. Mülleder M. Hemmati-Sadeghi S. Klein C.. (2021). Dietary-challenged mice with Alzheimer-like pathology show increased energy expenditure and reduced adipocyte hypertrophy and steatosis. Aging (Albany NY) 13, 10891–10919. doi: 10.18632/aging.202978, PMID: 33864446 PMC8109068

[ref52] Shoji H. Miyakawa T. (2019). Age-related behavioral changes from young to old age in male mice of a C57BL/6J strain maintained under a genetic stability program. Neuropsychopharmacol. Rep. 39, 100–118. doi: 10.1002/npr2.12052, PMID: 30816023 PMC7292274

[ref53] Singh B. Olds T. Curtis R. Dumuid D. Virgara R. Watson A.. (2023). Effectiveness of physical activity interventions for improving depression, anxiety and distress: an overview of systematic reviews. Brit J Sport Med. 57, 1203–1209. doi: 10.1136/bjsports-2022-106195, PMID: 36796860 PMC10579187

[ref54] Soto J. E. Burnett C. M. L. Ten Eyck P. Abel E. D. Grobe J. L. (2019). Comparison of the effects of high-fat diet on energy flux in mice using two multiplexed metabolic phenotyping systems. Obesity 27, 793–802. doi: 10.1002/oby.22441 30938081 PMC6478533

[ref55] Stojakovic A. Walczak M. Cieślak P. E. Trenk A. Sköld J. Zajdel J.. (2018). Several behavioral traits relevant for alcoholism are controlled by ɣ2 subunit containing GABA(a) receptors on dopamine neurons in mice. Neuropsychopharmacology 43, 1548–1556. doi: 10.1038/s41386-018-0022-z, PMID: 29463910 PMC5957272

[ref56] Tatem K. S. Quinn J. L. Phadke A. Yu Q. Gordish-Dressman H. Nagaraju K. (2014). Behavioral and locomotor measurements using an open field activity monitoring system for skeletal muscle diseases. J. Vis. Exp. 91:51785. doi: 10.3791/51785 PMC4672952 25286313

[ref57] Terry S. Gommet C. Kerangueven A. C. Leguet M. Thévenin V. Berthelot M.. (2023). Activity in group-housed home cages of mice as a novel preclinical biomarker in oncology studies. Cancers 15:194798. doi: 10.3390/cancers15194798, PMID: 37835492 PMC10571829

[ref58] VanWormer J. J. Boucher J. L. Sidebottom A. C. Sillah A. Knickelbine T. (2017). Lifestyle changes and prevention of metabolic syndrome in the heart of New Ulm project. Prev. Med. Rep. 6, 242–245. doi: 10.1016/j.pmedr.2017.03.018 28377851 PMC5377429

[ref59] Wermelinger Ávila M. P. Corrêa J. C. Zaidem M. Passos M. V. Sena Lomba Vasconcelos A. P. Lucchetti A. L. G.. (2022). Resilience and mental health among regularly and intermittently active older adults: results from a four-year longitudinal study. J. Appl. Gerontol. 41, 1924–1933. doi: 10.1177/07334648221095075, PMID: 35543168

[ref60] Zentrich E. Talbot S. R. Bleich A. Häger C. (2021). Automated home-cage monitoring during acute experimental colitis in mice. Front Neurosci-Switz. 15:15. doi: 10.3389/fnins.2021.760606 PMC8570043 34744621

[ref61] Zhang X. Y. Ye F. Yin Z. H. Li Y. Q. Bao Q. N. Xia M. Z.. (2024). Research status and trends of physical activity on depression or anxiety: a bibliometric analysis. Front Neurosci-Switz. 18:18. doi: 10.3389/fnins.2024.1337739 PMC10996447 38586196

